# Circulating microRNAs in Carotid Atherosclerosis: Complex Interplay and Possible Associations with Atherothrombotic Stroke

**DOI:** 10.3390/ijms251810026

**Published:** 2024-09-18

**Authors:** Marine M. Tanashyan, Alla A. Shabalina, Vladislav A. Annushkin, Andrey S. Mazur, Polina I. Kuznetsova, Anton A. Raskurazhev

**Affiliations:** 1 Research Center of Neurology, 80, Volokolamskoe Shosse, 125367 Moscow, Russiaannushkin@neurology.ru (V.A.A.); kuznetsova@neurology.ru (P.I.K.); 2 Laboratory of Hemorheology, Hemostasis and Pharmacokinetics with Clinical Laboratory Diagnostics, Research Center of Neurology, 80 Volokolamskoye Shosse, 125367 Moscow, Russia

**Keywords:** microRNA, cerebrovascular disease, carotid atherosclerosis, biomarkers, stroke

## Abstract

Atherosclerosis is a chronic inflammatory disorder which remains the main cause of cardiovascular morbidity and mortality, with carotid atherosclerosis (CA) being a major cause of ischemic stroke. Epigenetic regulation plays a significant role in CA progression and stroke, yet the impact of circulating microRNA expression, associated with atherogenesis, has not been clearly defined. We included 81 patients with moderate–severe CA (mean age 67 ± 7 years, 53% male), 42% of whom had prior ipsilateral ischemic stroke (i.e., were symptomatic). A total of 24 miRs were identified and their plasma expression levels were measured. We observed that several microRNAs were up-regulated in stroke survivors, namely miR-200c-3p (30.6 vs. 29.7, *p* = 0.047), miR-106b-3p (31.01 vs. 30.25, *p* = 0.004), and miR-494-5p (39 vs. 33, *p* < 0.001), while others (miR183-3p [25.5 vs. 28.6, *p* < 0.001], miR-126-5p [35.6 vs. 37.1, *p* = 0.03], and miR-216-3p [12.34 vs. 16.2, *p* < 0.001]) had lower plasma levels in symptomatic patients. In a multivariable logistic regression model for symptomatic CA, the only miRs showing statistical significance were miR-106b-5p, miR-183-3p, miR-216-3p, and miR-494-5p. Cluster analysis demonstrated differential miR expression in CA patients depending on their stroke status. Epigenetic modulation, represented as complex interplay between circulating miRs of different atherogenic potential, may play a significant role in CA development and progression. In our study, we show possible candidates for future research regarding CA and stroke.

## 1. Introduction

Carotid atherosclerosis (CA) remains a major cause of ischemic stroke, comprising up to 20% of stroke cases [1]. Atherosclerosis is a chronic inflammatory disorder which involves the vessel wall of large and medium arteries, associated with the accumulation of lipids (forming plaques) and leading to the narrowing of their lumen (stenosis) and subsequent thrombosis [2].

The prevalence of CA in the general population is estimated to be >1.1 billion cases, with carotid stenosis (>50%) constituting 1.5% (approximately 58 million cases) [3]. The risk of stroke increases with the degree of carotid artery stenosis [4]; significant CA refers to a >50% narrowing of the carotid artery, with the term ‘symptomatic CA’ applied to patients who have ipsilateral stroke/transient ischemic attack. However, the degree of stenosis appears to be not sufficient enough to accurately predict stroke risk—nearly half of strokes in the pivotal surgical trials for CA (NASCET [North American Symptomatic Carotid Endarterectomy Trial] and ECST [European Carotid Surgery Trial]) occurred in patients with <50% carotid stenosis [5]. Atherosclerotic plaque vulnerability (i.e., a ‘stable’ vs. ‘unstable’ phenotype) may be a promising surrogate marker, better at identifying high-risk asymptomatic patients for whom aggressive treatment would be beneficial.

Epigenetic regulation plays a significant role in atherosclerosis initiation and progression with non-coding RNAs as potential identifiable blood biomarkers [6]. MicroRNAs are small (20–40 nucleotides), relatively stable, and conserved molecules highly involved in atherogenesis [7]. Previously [8], we described a panel of microRNA involved in various pathological processes underlying atherosclerosis: inflammation, endothelial dysfunction, lipid/cholesterol metabolism, oxidative stress, the regulation of angiogenesis, and smooth muscle cell proliferation. In this study, we aimed to identify possible epigenetic markers (via circulating microRNA) of CA and elucidate their association with ischemic stroke.

## 2. Results

A brief summary of patient characteristics is presented in Table 1. There were no statistically significant differences between symptomatic and asymptomatic patients with CA by demographic and main clinical parameters. The median carotid stenosis levels were 75% in both groups, which corresponded to advanced CA. Nearly all patients had a history of arterial hypertension, and one-third had diabetes. Interestingly, low-density lipoprotein (LDL) levels were within the target range (<1.8 mmol/L) in patients with prior stroke, which could be explained by a potentially higher proportion of statin users in this group.

**Figure 1:**
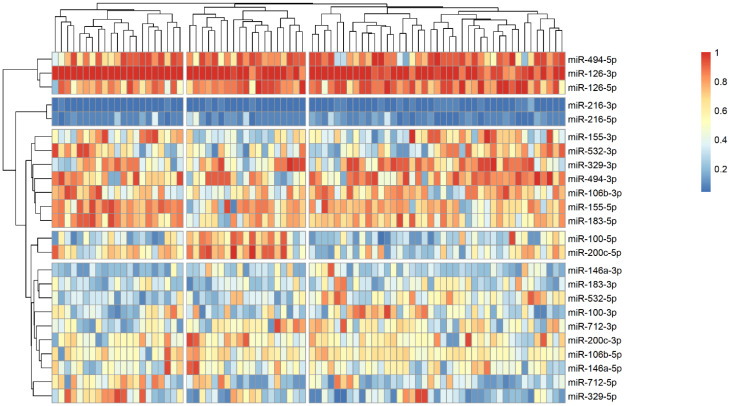
Heatmap showing relative microRNA levels in all patients with CA; clusters visualized via hierarchical clustering. Columns represent individual patients, rows—relative miR expression (with larger values on the color legend corresponding to higher relative levels). Dendrograms were calculated via the optimal leaf ordering algorithm; both row and column clusters are separated by narrow white spaces.

**Figure 2:**
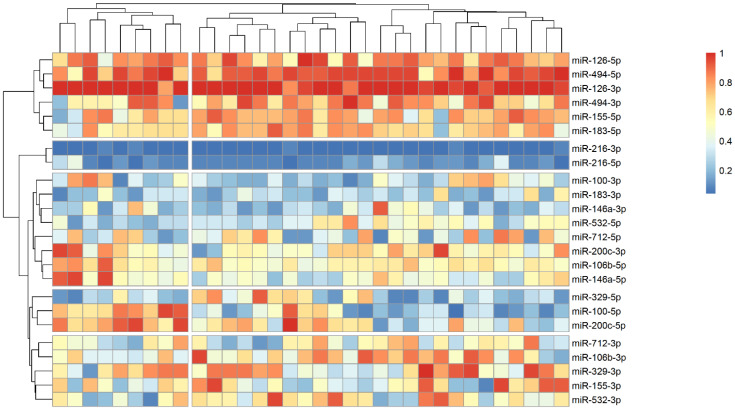
Heatmap showing relative microRNA levels in stroke CA patients; clusters visualized via hierarchical clustering. Columns represent individual patients, rows—relative miR expression (with larger values on the color legend corresponding to higher relative levels). Dendrograms were calculated via the optimal leaf ordering algorithm; both row and column clusters are separated by narrow white spaces.

**Figure 3:**
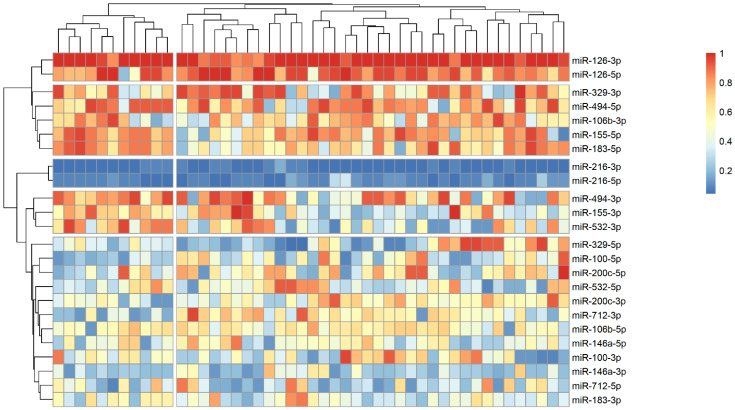
Heatmap showing relative microRNA levels in asymptomatic CA patients; clusters visualized via hierarchical clustering. Columns represent individual patients, rows—relative miR expression (with larger values on the color legend corresponding to higher relative levels). Dendrograms were calculated via the optimal leaf ordering algorithm; both row and column clusters are separated by narrow white spaces.

The analysis of microRNA expression between groups revealed several statistically significant differences (Table 2). We observed that several microRNAs are up-regulated in stroke survivors, namely miR-200c-3p, miR-106b-3p and miR-494-5p, while others (miR183-3p, miR-126-5p and miR-216-3p) have lower plasma levels in symptomatic patients. In order to identify possible clusters of similar microRNA expression, we performed a hierarchical clustering analysis visualized by heatmaps (Figure 1, Figure 2 and Figure 3). When looking at all patients with CA, we see a cluster of almost uniformly up-regulated (miR-494-5p and miR-126-5p/3p) and down-regulated (miR-216-5p/-3p) microRNAs, with all others showing different expression across clusters. In symptomatic CA patients (Figure 2), the cluster of up-regulated microRNAs shows several more markers (i.e., miR-155-5p and miR-183-5p), while asymptomatic CA patients (Figure 3) show two more clusters of up-regulated microRNAs (namely miR-155-3p, miR-532-3p). MiR-100-5p and miR-200c-5p demonstrate overexpression in a cluster of patients (Figure 1, middle group of columns), corresponding to symptomatic CA patients.

**Figure 4:**
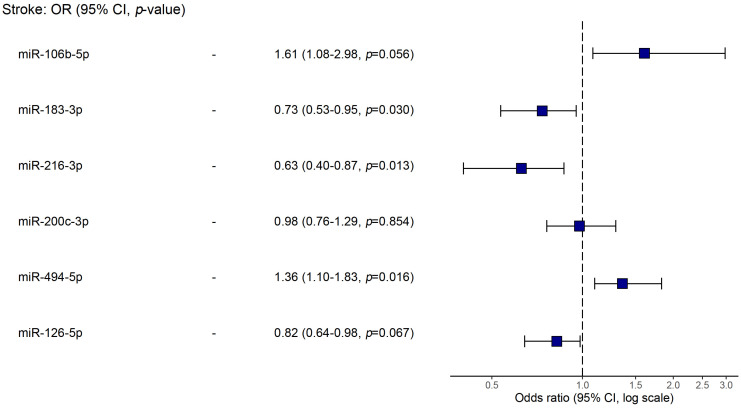
Forest plot of the multivariable logistic regression for associations with stroke.

Using logistic regression, we analyzed possible associations of studied biomarkers (microRNAs, level of stenosis, age and LDL levels) with stroke (Table 3). In a model of univariable regression, we found that the up-regulation of miR-106b-5p, miR-200c-3p, and miR-494-5p and the down-regulation of miR-183-3p, miR-216-3p, and miR-126-5p were linked to stroke occurrence (*p* < 0.05). Yet, in a multivariable model, the only miRs showing statistical significance were miR-106b-5p, miR-183-3p, miR-216-3p, and miR-494-5p. The latter are plotted in Figure 4.

**Figure 5:**
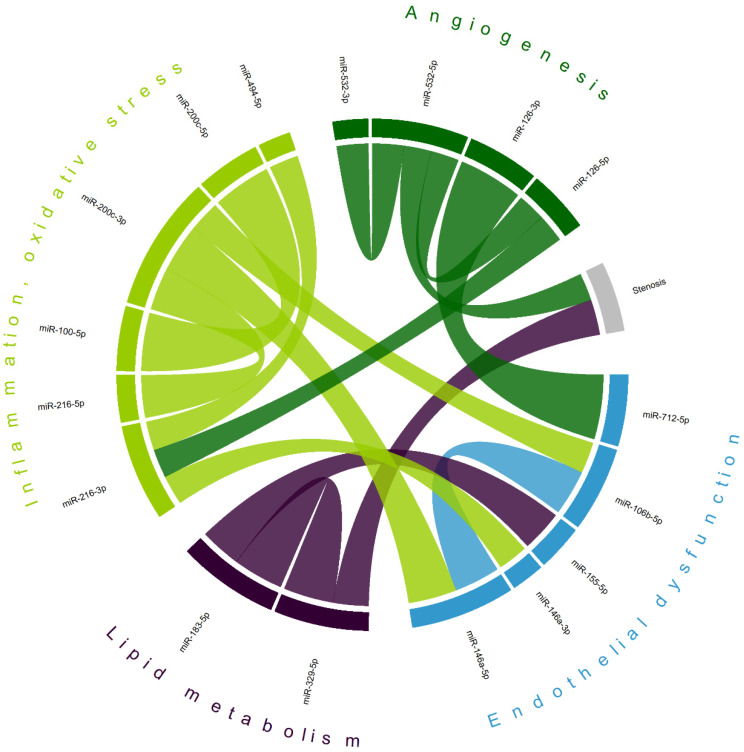
Chord diagram of most correlated microRNAs; matching colors represent groups of microRNAs primarily associated with different mechanisms of atherogenesis. Shown are only statistically significant correlations with the absolute value of Pearson’s coefficient > 0.3; most correlations are positive (exceptions—stenosis/miR-532-5p and miR-216-3p/miR-494-5p); the width of each chord is proportional to Pearson’s coefficient.

Correlation analysis (Table S1) has demonstrated a complex interplay between various microRNAs in patients with CA (Figure 5), not only within clusters of pathological processes involved in atherosclerosis, but also between clusters. The levels of two microRNAs were found to be correlated with carotid stenosis measurements (miR-329-5p [r = 0.391] and miR-532-5p [r = −0.303]), which may indicate their potential as biomarkers of CA progression. The largest absolute correlation was observed between miR-126-3p (of the ‘angiogenesis’ domain) and miR-712-5p (associated with ‘endothelial dysfunction’), r = 0.713. A previously described cluster of overexpressed miR-183-5p and miR-155-5p in CA stroke patients also shows a moderate positive correlation (r = 0.456). Intradomain correlations, including miR-100-5p and miR-200c-5p (r = 0.643, also observed as a separate cluster in symptomatic CA patients)—miRs involved in inflammation and oxidative stress—reveal a complex interplay of various factors associated with atherogenesis.

In order to identify possible ‘miR’–‘target gene’ interactions, we performed a network analysis via MIENTURNET (see ‘Statistics’ section), limiting the threshold for the minimum number of miRNA–target interactions to three and the FDR to 0.1 (Figure S1). A number of common target genes were observed, including *PHF14, UBQLN1, MYLK, HIF1A*, *FLT1*, *PTEN*, *KRAS*, *SIRT1*, *PDCD4*, *CXCR4*, *TCF4*, and *CXCL12*.

## 3. Discussion

Epigenetic regulation plays an important role in atherosclerotic cerebrovascular disease, with microRNAs as potential biomarkers for CA progression and stroke occurrence. In our study, we show that the differentiated expression of certain miRNA clusters may affect stroke susceptibility in CA patients with hemodynamically significant carotid stenosis.

miR-200c-3p plasma levels were higher in atherothrombotic stroke survivors—this is a microRNA closely related to (and up-regulated by) oxidative stress and reactive oxygen species formation [9]. The higher expression of miR-200c in stroke models [10], given a possible neuroprotective effect of anti-miR-200c treatment [11], may suggest a putative role of this microRNA in ischemic stroke. It has also been regarded as a promising biomarker of carotid atherosclerotic plaque instability [12], possibly mediated by promoting oxidized LDL-induced endothelial to mesenchymal transition [13]. Both miR-200c-3p and miR-100-5p have the *FLT1* gene as a predicted target—this gene is closely involved in vascular integrity and is regarded as a candidate atherosclerosis gene [14].

Stroke patients with CA had up-regulated levels of miR-106b-5p—a microRNA which was also positively correlated with miR-200c-3p. In a study of patients with atherosclerosis, miR-106b levels were higher compared to control patients [15]; stroke patients had consistent elevation of miR-106b (according to [16]). Antagonism to miR-106b in a mouse model of stroke proved to be neuroprotective [17]. A common target of miR-106b-5p and miR-155-5p is hypoxia-induced factor 1A gene (*HIF1A*), which is regarded as proatherogenic [18].

Another significantly up-regulated microRNA in CA stroke patients was miR-494-5p—an miR highly expressed in unstable carotid atherosclerosis plaques [19]. Interestingly, miR-494 is a part of the 14q32 miR gene cluster, the hypomethylation of which has been shown to be implicated with the development of atherosclerosis [20]. miR-494 targets inflammatory genes, and its inhibition in mice resulted in a reduction in atherosclerotic lesions and an increase in plaque stability (including via an increase in collagen content) [21]. Circulating miR-494 was elevated in patients with acute ischemic stroke, which corroborates our current findings; moreover, it was a predictor of poor long-term prognosis [22].

Several miRs were down-regulated in CA stroke patients—notably, miR 183-3p, which had a different expression profile than its 5p counterpart. Experimental data show that miR-183-3p may regulate the proliferation of vascular smooth muscle cells binding to KCNQ1OT1, a long-coding RNA, up-regulated in atherosclerosis [23]. In the stroke setting, a possible role for miR-183 lies in negatively regulating *PTEN* gene expression—it has been shown that following ischemia in mice, the levels of miR-183 were reduced, while transfection with agomiR-183-5p resulted in reduced cerebral ischemic injury and apoptosis [24]. Another important target for miR-183 is *FOXO1*, which has been found to be associated with cell apoptosis, cell cycle progression, and oxidative stress—a study has shown the mediating role of miR-183 in cerebral ischemia–reperfusion injury [25].

Another down-regulated microRNA was miR-216, which plays an important role in vascular endothelial senescence, inflammation, and macrophage regulation in atherosclerosis [26]. The plasma expression of miR-216 was higher in unstable coronary disease patients [27], and in CA, it was demonstrated that miR-216 may promote endothelial inflammation and monocyte adhesion via the Smad7/IκBα pathway [28]. However, in stroke models, the up-regulation of miR-216 is shown to be beneficial for inducing neuroprotection against cerebral ischemia by targeting *JAK2* [29]. This latter hypothesis agrees with our data where stroke patients had significantly lower levels of circulating miR-216. But overall, in our cohort of patients, miR-216-3p/-5p were the most underexpressed miR (as visualized by heatmap as shown in Figure 1, Figure 2 and Figure 3), forming a cluster of their own. Along with miR-200c-3p, they share an important anti-inflammatory target gene—*SIRT1*—which regulates endothelial activation, platelet aggregation and coagulation, along with oxidative stress and thrombosis [30].

Interestingly, most miRs demonstrated positive correlations between each other, with only one—miR-535-5p—negatively associated with carotid stenosis degree. In a study by Sun H. et al. [31], serum miR-532-5p was inversely related to carotid intima media thickness, which corroborates our findings. The authors showed that, in vitro, the overexpression of miR-532-5p inhibited the proliferation and migration of vascular smooth muscle cells—a crucial step in atherogenesis. Consequently, patients with more severe CA exhibited lower plasma levels of circulating miR-532-5p. Among other targets, miR-532-5p is linked to programmed cell death 4 (*PDCD4*) gene expression—which acts as a regulator of apoptosis in vascular smooth muscle cells [32]. This gene is also a target for miR-183-5p, another highly expressed miR in CA patients.

Our target interaction analysis revealed several possible mediators involved in CA; e.g., miR-155-5p and miR-200c-3p share two significant target genes—*MYLK* (myosin light chain kinase) and *UBQLN1* (ubiquilin-1). The former is one of the key regulators of endothelial cytoskeletal organization [33] and thus may be involved in endothelial dysfunction and atherosclerosis initiation. The latter is closely related to ischemic stroke pathophysiology as ubiquitin seems to be protective against oxidative stress and ischemic/reperfusion injury [34]. A protooncogene—*KRAS* (Kirsten rat sarcoma virus)—was found to be a target of miR-126-3p, miR-200c-3p, and miR-155-5p, confirming emerging evidence of its critical role in atherosclerosis and ischemic stroke [35]. We also elucidated a cluster of chemokine signaling regulation via microRNAs involving *CXCL12* and *CXCR4* (miR-126-5p/-3p, miR-146a-5p/-3p and miR-494-5p)—factors closely associated with atherosclerosis development and progression [36].

## 4. Materials and Methods

### 4.1. Study Population

We included in this cross-sectional prospective study 81 consecutive patients (mean age 67 ± 7 years, 53% male) with carotid atherosclerosis (CA) admitted to the angioneurology department of our clinic (Research Center of Neurology, Moscow, Russia) during the period of 2023–2024. All patients had moderate–advanced carotid stenosis (>50%), and 42% were symptomatic (i.e., had prior [>6 months] ipsilateral ischemic stroke). We excluded patients with decompensated somatic (e.g., cardiovascular, renal, hepatic) disease, a history or current signs of neoplasm, concurrent infectious, autoimmune or rheumatic disorder, or those who had undergone major surgery less than 3 months prior. All patients gave informed consent.

All patients underwent a thorough clinical and neurological examination, routine blood analyses (including low-density lipoproteins [LDLs] and total cholesterol [TC]), ultrasound examination of the extracranial carotid arteries (with luminal stenosis measured according to the NASCET criteria [37]).

### 4.2. Laboratory Analysis

Blood collection was carried out in the morning, on an empty stomach in vacutest tubes (with clot activator). Since it is known from the literature that the level of microRNAs can increase due to hemolysis, serum samples with its presence were not used [38].

A total of 24 microRNAs were analyzed (hsa-miR-200c-3p, hsa-miR-200c-5p, hsa-miR-146a-3p, hsa-miR-146b-5p, hsa-miR-155-3p, hsa-miR-155-5p, hsa-miR-106b-3p, hsa-miR-106b-5p, hsa-miR-183-3p, hsa-miR-183-5p, hsa-miR-126-3p, hsa-miR-126-5p, hsa-miR-712-3p, hsa-miR-712-5p, hsa-miR-532-3p, hsa-miR-532-5p, hsa-miR-494-3p, hsa-miR-494-5p, hsa-miR-329-3p, hsa-miR-329-5p, hsa-miR-100-3p, hsa-miR-100-5p, hsa-miR-216-3p, hsa-miR-216-5p).

RNA (including micro–RNA) was isolated from 200 µL of serum using the miRNeasy Serum/Plasma Advanced Kit—microRNA Isolation (Qiagen, Hilden, Germany) in accordance with the manufacturer’s protocol. The RNA concentration was evaluated using a Qubit 4 fluorimeter (Thermo Fisher Scientific, Waltham, MA, USA). Exogenous synthetic cel-miR-39-3p (0.2 nM long microRNA *C. elegans*) was used to normalize microRNA levels. miR-16-5p was chosen as an endogenous normalizer of circulating microRNA expression due to its relative stability in serum/plasma shown by various studies and its widespread use as a reference in the association of circulating microRNAs with various pathologies [39,40,41,42].

RNA samples (final volume 20 µL) were stored at a temperature of −80 °C until the stage of cDNA synthesis in the reverse transcription reaction.

For the reverse transcription and determination of microRNA transcripts, sets of ALMIR reagents (Algimed Techno RT–PCR, Belarus, Russia) containing optimized enzyme mixtures for reverse transcription and PCR and balanced solutions of oligonucleotides for the analysis of a specific miRNA molecule were used: an RT primer, a pair of PCR primers, and a fluorescently labeled probe for amplification detection [43].

The volume of the microRNA sample for cDNA synthesis was 2 µL, in accordance with the manufacturer’s instructions.

The quantitative determination of microRNA transcripts was performed by real-time PCR using the CFX96 C1000 Touch amplifier (BioRad, California, CA, USA) according to the following program: activation of the enzyme—20 c at 95 °C; 45 cycles, denaturation—1 c at 95 °C, annealing/elongation—20 c at 60 °C. The relative microRNA level was calculated using the 2^−ΔΔCt^ method using the appropriate CFX Manager (version 3.1) software.

### 4.3. Statistical Analysis

Statistical analysis and visualization were performed using R programming language (version 4.4.1) in RStudio environment (version 2024.04.2), utilizing the following packages: ‘tidyverse’, ‘sjPlot’, ‘corrplot’, ‘finalfit’, ‘circlize’, ‘gtsummary’, ‘ggstatsplot’, and ‘pheatmap’. A comparison of continuous variables between two independent groups was performed using the Wilcoxon–Mann–Whitney test (rank-sum test), and for discrete variables, we used Pearson’s Chi-squared test or Fisher’s exact test. The Pearson correlation coefficient was used to identify possible associations between continuous variables; then, the matrix of coefficients was further visualized via a chord diagram using only statistically significant correlations with the absolute value of the coefficient > 0.3. Logistic regression (first univariable, then multivariable) was performed with ‘stroke’ as the dependent variable and most continuous variables as independent (explanatory). A heatmap using the expression of microRNAs was constructed with clustering using the ‘optimal leaf ordering’ algorithm (https://www.mathworks.com/help/stats/optimalleaforder.html; accessed on 1 August 2024); relative expression was calculated using the ‘percentize’ function; the color scheme represents relative expression; columns are individual cases. Missing data were treated as missing at random; they were dealt with according to the CART (classification and regression trees) method of the ‘mice’ package. Possible ‘MiR’–‘target’ interactions were analyzed using the MIENTURNET tool [44] with the miRTarBase database. All statistical tests were two-sided; the alpha-level was 0.05.

## 5. Conclusions

For the first time (to our knowledge), a broad spectrum of circulating microRNAs associated with atherogenesis was analyzed in a cohort of moderate–severe carotid atherosclerosis patients (both symptomatic and asymptomatic). A number of miRs (incl. miR-183, miR-216, miR-106b, and miR-494) was independently associated with symptomatic carotid stenosis; they may be chosen as key markers for future prospective studies. CA patients exhibit a differential expression pattern of studied miRs based on their stroke status. Epigenetic modulation, represented as complex interplay between circulating miRs of different atherogenic potential, may play a significant role in CA development and progression.

## 6. Limitations

The study has several limitations, of which its design (no follow-up) and relatively small sample size may be the most important. Both arose due to the pilot nature of this study—in most studies on microRNAs (in patients), the research is primarily focused on a handful of microRNAs, and the samples are usually not as large as for other biomarker studies. In our work, we decided to analyze a large number of potentially relevant microRNAs with the goal of identifying the most promising ones for future research. The choice of the microRNA panel presents potential bias as we probably may have omitted many relevant microRNAs—in our previous manuscript, we outlined the methodology underlying our strategy, but, as miRs have multiple targets, their partition (i.e., as in Figure 5) is largely arbitrary and serves a more graphic purpose. Selection bias arose due to the single-center nature of this study and may be a significant confounding factor (firstly, because severe stroke associated with CA has high mortality rates, and thus, these patients could not have been included; secondly, because most patients were on the best medical treatment for CA, which could have influenced miR expression). No in vitro or in vivo experiments were possible to confirm possible miRNA–target interactions, which prompted us to use publicly available databases, which is a limitation.

## Figures and Tables

**Table 1:** Patient characteristics.

	Patients with CA (n = 81)		
Parameter	Asymptomatic ^1^ (n = 47)	Symptomatic ^1^ (n = 34)	*p*-Value ^2^
Age, yrs	69 (61–74)	67 (60–69)	0.21
Gender			0.38
Female	24 (51)	14 (41)	
Male	23 (49)	20 (59)	
Carotid stenosis, %	75 (70–85)	75 (70–80)	0.36
AH	46 (98)	34 (100)	>0.99
DM	13 (28)	12 (35)	0.46
LDL, mmol/L	1.96 (1.55–2.64)	1.72 (1.30–2.15)	0.087
TC, mmol/L	4.95 (4.20–5.90)	4.20 (3.80–5.80)	0.19

^1^ Median (IQR); n (%); ^2^ Wilcoxon rank sum test; Pearson’s Chi-squared test; Fisher’s exact test; AH—arterial hypertension, DM—diabetes mellitus, LDL—low-density lipoprotein, TC—total cholesterol.

**Table 2:** MicroRNA expression depending on stroke occurrence.

	Patients with CA		
miRs	Asymptomatic ^1^	Symptomatic ^1^	*p*-Value ^2^
miR-200c-3p	29.7 (28.4, 31.5)	30.6 (29.4, 33.1)	0.047
miR-200c-5p	29.8 (25.4, 33.0)	32.6 (28.2, 35.1)	0.082
miR-146a-3p	26.1 (24.5, 28.2)	26.4 (24.1, 28.1)	>0.9
miR-146a-5p	28.9 (27.8, 29.9)	29.9 (28.0, 30.8)	0.2
miR-155-3p	29.6 (26.9, 33.0)	29.7 (26.8, 34.5)	0.8
miR-155-5p	33.5 (32.2, 34.4)	33.1 (31.6, 34.5)	>0.9
miR-106b-3p	31.7 (29.4, 34.3)	31.3 (28.4, 35.3)	0.9
miR-106b-5p	30.25 (29.80, 30.97)	31.01 (30.26, 31.51)	0.004
miR-183-3p	28.6 (26.5, 30.1)	25.5 (22.1, 28.3)	<0.001
miR-183-5p	32.7 (29.7, 34.4)	32.6 (31.1, 34.3)	>0.9
miR-126-3p	45 (41, 52)	50 (43, 57)	0.073
miR-126-5p	37.1 (34.8, 42.1)	35.6 (33.9, 37.0)	0.030
miR-712-3p	30.0 (26.6, 31.0)	30.2 (26.5, 31.2)	0.9
miR-712-5p	26.9 (23.7, 30.9)	29.1 (24.5, 33.2)	0.12
miR-532-3p	29.7 (26.0, 34.1)	31.2 (27.4, 33.1)	0.5
miR-532-5p	28.7 (26.3, 30.6)	28.2 (26.3, 29.8)	0.3
miR-494-3p	33.5 (29.6, 37.7)	34.6 (31.4, 37.8)	0.3
miR-494-5p	33 (31, 38)	39 (36, 44)	<0.001
miR-329-3p	32.9 (29.6, 38.8)	33.2 (28.5, 36.7)	0.5
miR-329-5p	28 (24, 30)	27 (25, 31)	0.7
miR-100-3p	28.8 (25.4, 31.1)	27.1 (24.0, 29.8)	0.14
miR-100-5p	28.4 (23.9, 30.6)	28.5 (23.1, 36.6)	0.5
miR-216-3p	16.23 (14.29, 18.23)	12.34 (11.24, 15.12)	<0.001
miR-216-5p	18.3 (16.9, 20.3)	18.1 (16.4, 20.0)	>0.9

^1^ Median (Q1, Q3); ^2^ Wilcoxon rank sum exact test; Wilcoxon rank sum test.

**Table 3:** Regression analysis for factors associated with symptomatic CA.

Dependent: Stroke	OR (Univariable)	OR (Multivariable)
Stenosis	1.00 (0.97–1.03, *p* = 0.956)	-
Age	0.97 (0.92–1.02, *p* = 0.230)	-
LDL	0.57 (0.28–1.04, *p* = 0.080)	-
miR-712-5p	1.08 (0.99–1.19, *p* = 0.091)	-
miR-712-3p	1.00 (0.89–1.13, *p* = 0.982)	-
miR-106b-3p	1.01 (0.91–1.12, *p* = 0.831)	-
miR-106b-5p	1.49 (1.12–2.41, *p* = 0.037)	1.61 (1.08–2.98, *p* = 0.056)
miR-155-5p	1.00 (0.90–1.10, *p* = 0.967)	-
miR-155-3p	1.00 (0.92–1.09, *p* = 0.965)	-
miR-146a-3p	0.99 (0.85–1.14, *p* = 0.870)	-
miR-146a-5p	1.09 (0.98–1.25, *p* = 0.135)	-
miR-329-3p	0.97 (0.90–1.04, *p* = 0.413)	-
miR-329-5p	0.98 (0.91–1.05, *p* = 0.542)	-
miR-183-3p	0.75 (0.63–0.87, *p* = 0.001)	0.73 (0.53–0.95, *p* = 0.030)
miR-183-5p	0.96 (0.85–1.08, *p* = 0.499)	-
miR-216-3p	0.67 (0.52–0.82, *p* < 0.001)	0.63 (0.40–0.87, *p* = 0.013)
miR-216-5p	1.01 (0.89–1.15, *p* = 0.879)	-
miR-100-3p	0.93 (0.84–1.02, *p* = 0.153)	-
miR-100-5p	1.02 (0.94–1.10, *p* = 0.616)	-
miR-200c-3p	1.13 (1.01–1.29, *p* = 0.042)	0.98 (0.76–1.29, *p* = 0.854)
miR-200c-5p	1.08 (0.99–1.19, *p* = 0.075)	-
miR-494-3p	1.05 (0.97–1.15, *p* = 0.253)	-
miR-494-5p	1.23 (1.12–1.39, *p* < 0.001)	1.36 (1.10–1.83, *p* = 0.016)
miR-532-3p	1.02 (0.94–1.12, *p* = 0.617)	-
miR-532-5p	0.94 (0.83–1.06, *p* = 0.310)	-
miR-126-3p	1.06 (1.00–1.13, *p* = 0.054)	-
miR-126-5p	0.89 (0.80–0.98, *p* = 0.026)	0.82 (0.64–0.98, *p* = 0.067)

OR—odds ratio; - = not included in the multivariable regression model.

## Data Availability

The data that support the findings of this study are available from the corresponding author upon reasonable request.

## Supplementary Materials

The following supporting information can be downloaded at: https://www.mdpi.com/article/10.3390/ijms251810026/s1.
