# Supplementary figures and images for: Circulating microRNAs in Carotid Atherosclerosis: Complex Interplay and Possible Associations with Atherothrombotic Stroke
Source: Int J Mol Sci. 2024 Sep 18;25(18):10026. doi: 10.3390/ijms251810026 (PMC11432131; doi:10.3390/ijms251810026)

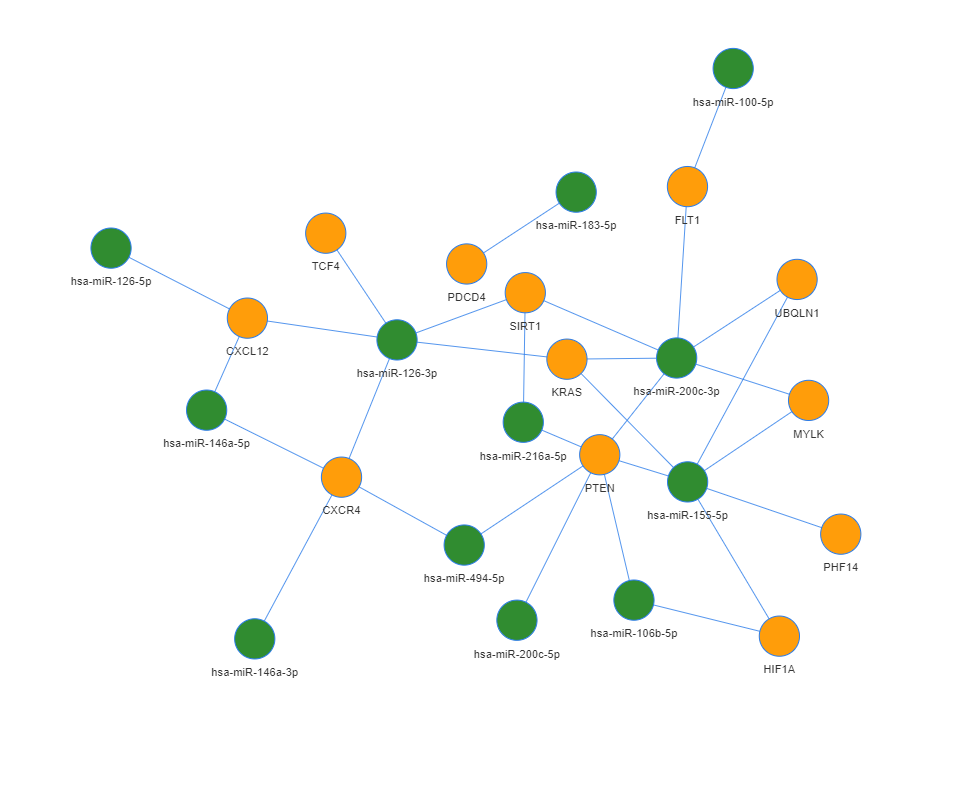

Supplement: Supplementary file 1 [file ijms-25-10026-s001.zip › FigureS1.png]
